# Emergent laparoscopy in treatment of perforated peptic ulcer: a local experience from a tertiary centre in Saudi Arabia

**DOI:** 10.1186/1749-7922-8-10

**Published:** 2013-03-08

**Authors:** Hamed Al Wadaani

**Affiliations:** 1 Department of Surgery, College of Medicine, King Faisal University & King Fahd Hospital, Al- Ahsa, KSA

**Keywords:** Perforated peptic Ulcer, Laparoscopy

## Abstract

**Background/ purpose:**

Perforated peptic ulcer (PPU) is still an existing disease that occurs frequently in the 21st century despite of the wide availability of antiulcer medication and Helicobacter eradication. The current study aimed to evaluate the hypothesis that its outcome might be improved by using the laparoscopy. The outcome of treatment in terms of complications, mortality and hospital stay with relevant to laparoscopy was analyzed.

**Patients and methods:**

This prospective descriptive study was carried on the period of 3 years from July 2009 till July 2012. All patients with acute abdominal pain that was clinically diagnosed as having perforated peptic ulcer were included. Excluded from this study were those patients with concomitant bleeding from the ulcer and evidence of gastric outlet obstructions. Also excluded were those with evidence of large perforation more than 10 mm and patients with symptoms of more than 36 h durations for fear of septic shock.

**Results:**

Forty seven patients were studied out of a total 53 PPU patients; they were 41 males and 6 females with the male to female ratio of 6.8:1. Their age ranged from 19 to 55 years with the mean age of 39.5 ± 8.6 years. Forty five patients were successfully treated by laparoscopy while only 2 cases that were early presented with signs of hypovolumic shock were converted into laparotomy due to severe bleeding. The mean hospital stay was 75 ± 12.6 h. Post operative complications included death of one patient in the postoperative period at the Intensive care unit (ICU) plus post operative fever in the 2 patients who underwent laparotomy and it was amenable to treatment.

**Conclusions:**

Laparoscopic repair of a perforated peptic ulcer is an amenable and feasible technique within the hands of experienced laparoscopic surgeon when the cases are early and properly diagnosed.

## Introduction

Although perforated peptic ulcer disease is a common surgical emergency and a major cause of death in elderly patient controversy still exist regarding its tools of management [1,2]. Helicobacter pylori (H.P.) eradication has led to a significant decline in peptic ulcer prevalence [3]. However, the number of patients requiring surgical intervention remains relatively unchanged [4,5]. Non operative treatment of perforated peptic ulcers was shown to be effective [6]. Nevertheless, the uncertainty in diagnosis, the potential delay for treatment in non responders, and the unreliable response in some patients make it difficult to be applied to all clinical situations.

Various surgical techniques had been attempted for the treatment of perforated peptic ulcer (PPU). These included stapled omental patch [7], gastroscopy aided insertion of the ligamentum teres [8], or omental plug [9]. Yet, these techniques were either used only in small case series or tend to have high rates of re-operation. Laparoscopic suture closure, initially reported in 1990 [10], was considered to be safe as the open approach. It offers some merits including shorter hospital stay, less postoperative pain, and pulmonary infection with earlier return to normal activities [11]. Currently, the two most commonly accepted laparoscopic procedures for PPU are simple closure with or without an omental patch to cover the repaired ulcer assuming that it may decrease the probability of leakage and provide a further sense of security. The current study was designated to review the results of performing laparoscopic repair of PPU at a single tertiary centre in Saudi Arabia.

## Patient and methods

This Prospective descriptive study was performed was carried in a period of 3 years from July 2009 till July 2012. All patients with acute abdominal pain that was diagnosed as perforated peptic ulcer were enrolled in the study. A formal written consent was obtained on each case based on our institute ethical committee recommendations.

Excluded from this study were those patients with concomitant bleeding from the ulcer and evidence of gastric outlet obstructions. Patients with Boey risk score of 3 or more were excluded from laparoscopic interventions as they underwent a laprotomy approach. The Boey risk scoring system, propose by Boey et al. in 1987 [12], is well known for stratification of high risk patients in PPU. Also excluded were those with repeated upper abdominal operations, sever profound shock, extreme age, bleeding tendency, or the ulcer that was suspected to be malignant. The collected demographic data were age, gender, American Society of Anesthesiologists Association Score (ASA), presence of shock, White blood cell (WBC) count, Boey risk factor and co-morbidities of the patients.

Major medical illness, preoperative shock, intra-operative findings such as the location and size of perforation, severity of abdominal cavity contamination were all reviewed. It was surgeon’s discretion to decide whether omental patch be added or not after the perforated ulcer was closed.

Patients underwent the first aid supportive methods of not taking anything orally (NPO), the insertion of a naso-gastric tube for gastric decompression. Intravenous fluids were initially administrated in the form of crystalloids (saline or ringer’s lactate solution). Intravenous antibiotics were given in the form of third generation cephalosporin’s as well as metronedazole.

Routine laboratory tests were done including a complete blood counting (CBC) with differential leucocytes’ count; serum amylase and lipase were carried out to exclude acute pancreatitis. Moreover, all patients underwent abdominal x-rays to aid in diagnosing peritonitis. In cases where the X-rays were not conclusive; computed tomography (CT) was applied.

### Laparoscopy

**Figure 1:**
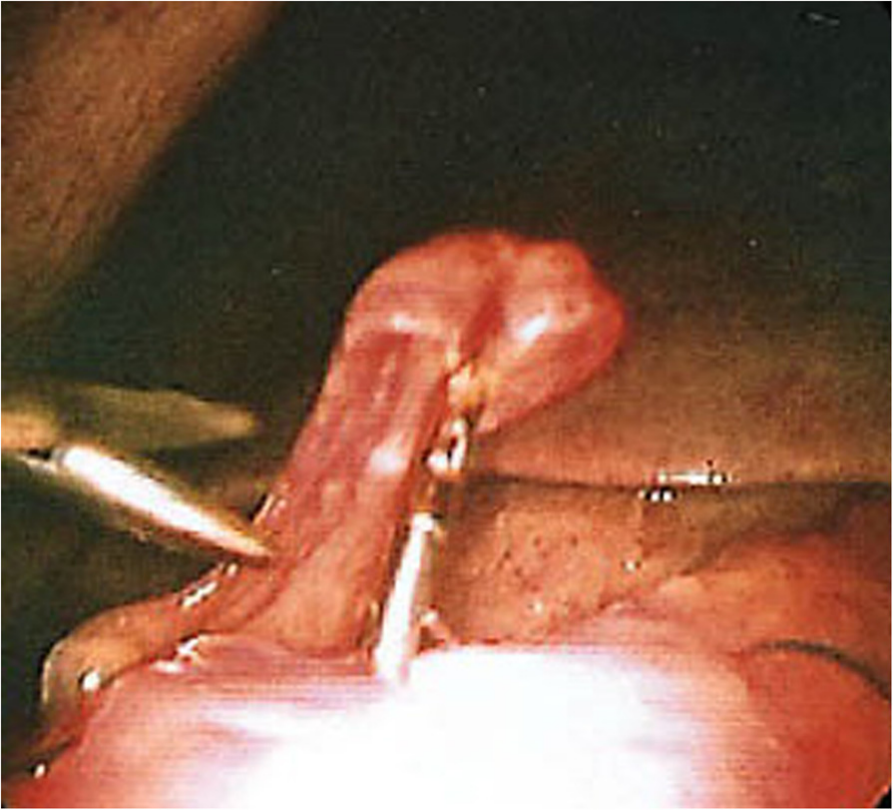
Laparoscopic photo of a perforated peptic ulcer (perforated 1jp).

**Figure 2:**
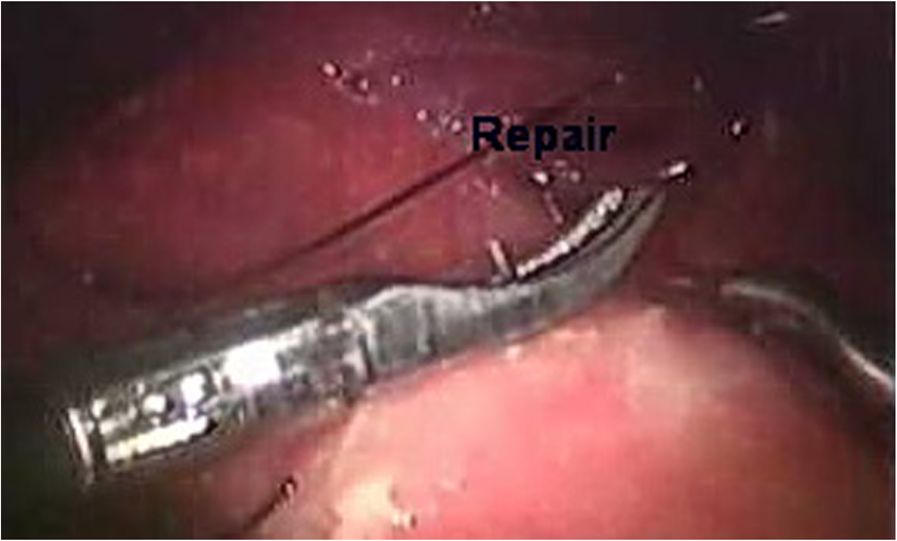
Laparoscopic photo of a direct suturing a perforated peptic ulcer (perf repair).

**Figure 3:**
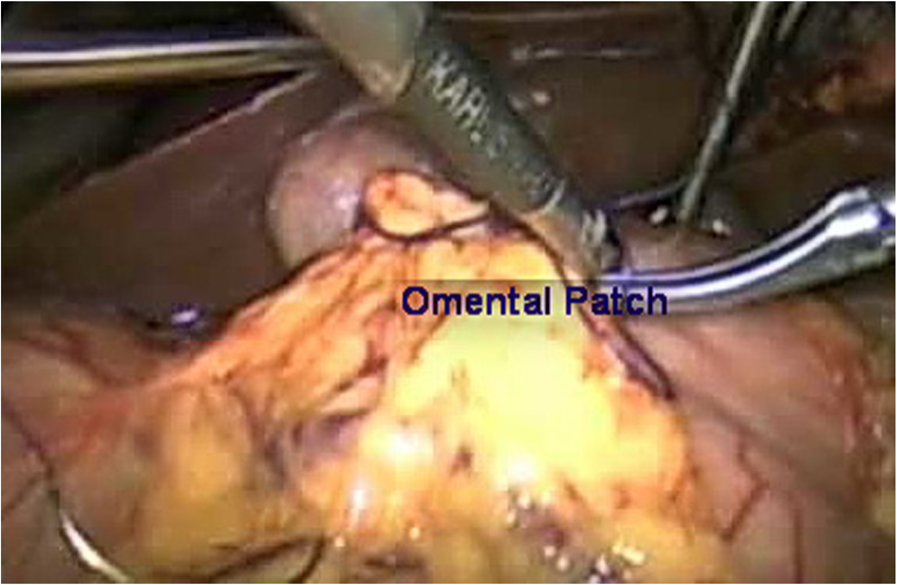
Laparoscopic photo of an omental patch.

All procedures were performed by the same senior consultant surgeon. In brief, patient was placed in a 15–20_ reverse Trendelenburg position. The operating surgeon stands to the patient’s left side. The periumbilical region is the usual site for initial access; however, in 2 patients with previous midline incisions dictated the use of another "virgin" site. Carbon dioxide pneumo-peritoneum with the insufflations pressure of 14–15 mmHg was applied in most cases; yet, we have used lower levels (8–12 mmHg) due to concerns of hemodynamic compromise with higher pressures in those patients with delayed onset of symptoms. An angled scope of A 10 mm 30_ laparoscope (Karl Storz, Tuttlingen Germany) was introduced through the umbilical 11 mm trocar (Versaport, Covidien Surgical Devices, North Haven, CT, USA) for inspection of the intra-abdominal organs, including the surface of the liver, gallbladder, stomach, intestine, pelvic organs, and visible retroperitoneal surfaces along with examination for free intraperitoneal fluid, followed by insertion of a second 11 mm trocar at left upper abdomen and another 5 mm trocar at right upper abdomen to optimize exposure or provide therapeutic intervention. Closing the perforated ulcer was done by using 3/0 polygalactin (Vicryl Ethicon, Johnson & Johnson, Cincinnati, OH, USA) stitches in interrupted fashion with intra-corporeal tie. The Omental patch was performed by mobilizing the greater omentum over the repaired ulcer and tie over by previous retained suture ends in buttressing manner (Figures 1, 2, 3).

The follow up period at the outpatient department (OPD) of those patients ranged from 4 to 24 months duration after being discharged from the hospital.

Collected data were coded, entered and statistically analyzesd using SPSS version 17. Variables of each group were reported as medians and interquartile ranges (IQR) whenever suitable. Two tailed tests of significance were used with confidence level of 95%. Discrete variables were expressed as counts and percentages. For continuous variables, we used mean and slandered deviations for reporting the data. *P* value of ≤ 0.05 was considered significant.

Serial Chi-square tests or Fisher exact tests were used to compare categorical variables wherever appropriate. Wilcoxon Rank Sum Test was used.

## Results

Forty seven (47) patients were included in this study out of 53 patients with acute abdominal pain that was diagnosed as having perforated peptic ulcer during a period of 3 years from July 2009 to July 2012. Six (6) patients were excluded out of the total 53 patients; 3 patients because of huge ugly scars of previous upper abdominal operations, 1 patient due to evidence of gastric outlet obstruction, and the remaining 2 because of concomitant sever ulcer bleeding (Table 1).

**Table 1:** Included and excluded patients

**Total patients’ number**	**Patients included in the study**	**Patients excluded of the study**
**53**	**47**	**Total = 6**
		Previous upper abdominal operations’ scars = **3**
		evidence of gastric outlet obstruction = **1**
		Concomitant ulcer bleeding **= 2**

The 47 patients who underwent laparoscopic approach were 41 males and 6 females with the male to female ratio of 6.8:1. Their age ranged from 19 to 55 years with the mean age of 39.5 ± 8.6 years. Most of patients (31 patients; 66%) were smokers. Yet, none of them gave a history of chronic use of drugs such as steroids while 23 patients (48.9%) gave history of over consumption of non steroidal anti-inflammatory drugs. No patients gave history of consuming any anti-peptic ulcer drugs.

The mean duration of symptoms was 11.5 ± 4.3 h. Forty five patients were successfully treated by laparoscopy while only 2 cases were converted into laparotomy due to sever intra operative bleeding. The mean hospital stay was 75 ± 12.6 h. Post operative complications included post operative fever in the 2 patients and it was amenable to treatment. One patient died in the postoperative period at the Intensive care unit (ICU). This patient belonged to ASA III group. He was expired because of multi organ failure; he had diabetes, hypertension, atrial fibrillation, nephropathy, thyrotoxicosis, and recent cerebrovascular accident.

The demographic characteristics of patients including age range, sex distribution, and American Society of Anesthesiology (ASA) classification status were recorded. The sites and sizes of ulcer perforations were also recorded. Also recorded were the preoperative characteristics such as duration of pain longer than 24 h, previous history of peptic ulcer disease, and recent consumption of non steroidal anti inflammatory drugs. No patient was reported to have a history of recent cocaine consumption. Boey score was also recoded reporting that major medical illness, preoperative shock, and longstanding perforation (more than 24 h) were considered poor prognostic factors. The results showed that hypotension could not reliably predict outcome, and all patients admitted with hypotension survived (Table 2).

**Table 2:** **Demographics of the studied patients with perforated peptic ulcer disease Total *(n *= 47)**

**Age (years, mean ±SD)**	**39.5 ± 8.6**	***n *= all**
Male (%)	87.2%	*n* = 41
Female (%)	12.8%	*n* = 6
History of NSAID use (%)	48.9%	*n* = 23 1,109
Smokers (%)	66%	*n* = 31
History of ulcer (%)	29.8%	*n* = 14
ASA I (%)	10.6%	*n* = 5
ASA II (%)	76.6%	*n* = 36
ASA III (%)	10.6%	*n* = 5
ASA IV (%)	2.1%	*n* = 1
Boey 0 (%)	14.8%	*n* = 7
Boey 1 (%)	65.9%	*n* = 31
Boey 2 (%)	17.2%	*n* = 8
Boey 3 (%)	2.1%	*n* = 1
Shock at admission (%)	4.3%	*n* = 2
Duration of symptoms (h)	11.5 ± 4.3	*n* = all
Free air on X-ray (%)	85%	*n* = 40
Symptoms >24 h (%)	8.5%	*n* = 4
Size perforation (mm)	5.5 ± 3.6	*n* = all
Hospital stay (hours, mean ±SD)	75 ± 12.6	*n* = all
*WBe* (mean ±SD)	12.3 ± 5.6	*n* = all
Localization ulcer		
Duodenal (%)	74.5%	*n* = 35
Juxtapyloric (%)	6.4%	*n* = 3
Gastric (%)	19.1%	*n* = 9
*WBe* white blood cells		

The mean laparoscopic repair operative time was 42 ± 16.7 min. Patients required significantly less parenteral analgesics that more than half of them did not ask for any pethidine injection. They had a lower visual analog pain score on postoperative days 1 and 3.

One patient early in this series had leakage after repair and required open drainage. Wound complications occurred in two converted patients in the laparoscopic group; one had a wound infection and the other had wound dehiscence. There were two patients with intra abdominal collections; one of them had leakage from the repaired site and required reoperation, and the other patient was managed by percutaneous drainage. Three patients were re operated on, one for leakage, another for gastric outlet obstruction, and the last one for wound dehiscence.

## Discussion

Advances in the medical treatment of peptic ulcer disease and *Helicobacter pylori* (H.P.) eradication have led to a significant decline in peptic ulcer prevalence and a dramatic decrease in the number of elective ulcer surgeries performed. Nonetheless, the number of patients requiring surgical intervention for complications such as perforations remains relatively unchanged [1,3,13-16].

Minimally invasive surgery has gained a highly expanding role in gastrointestinal surgery since the introduction of laparoscopic cholecystectomy. In the last few years, the role of laparoscopic surgery in management of perforated peptic ulcer has gained more popularity among laparoscopic gastrointestinal procedures [17-21]. Literature review showed some randomized trials highlighting the feasibility of laparoscopic repair of PPU [11,22-24]. Only a few literatures had reported patients’ series of more than 100 patients while some did emphasize results from subgroups of patients [25,26].

In our study of the 47 PPU patients it was evident during the operation that none of the patient had a diagnosis different from PPU. This discovery revealed the benefit of laparoscopy as a diagnostic procedure. These results can be compared to previously published data [27].

Conversion rate from laparoscopy to laparotomy was 4.3% (2/47) this may be compared to previously published data of a conversion rate of 8% (4/52) [28]. Moreover, it is also much lower compared to that reported in literature, where conversion rates as high as 60% were found [11,12,23]. This may be partially attributed to the experience and training of the laparoscopic surgeon who participated in this work, confirming the belief that this procedure should only be done by experienced surgeons [22,23,29]. In the current study, the mean Operating time was 42 ± 16.7. This can be considered as significantly shorter compared to previously published data in the literature for laparoscopy group of (75 min) [28], and also shorter than other reports in the literature [22,24].

A possible explanation for the shorter operative time is that laparoscopic suturing is easier especially if the edges of the perforation are not infiltrated and non friable [30,31]. Sutures easily tear out and it is more difficult to take large bites and to tie knots properly. In our series, the use of a single-stitch method described in the literature [25], fibrin glue, or a patch might have aided in shorting the mean operative time of the laparoscopic procedure [26-32]. Another reason for the decrease in operating time is that we did not perform the irrigation procedure in most of the cases. It was recorded that irrigation through a 5-mm or even a 10-mm trocar is time consuming, and suction of fluid decreases the volume of gas and reduces the pneumoperitoneum. There is no evidence that irrigation lowers the risk of sepsis [33]. We therefore have performed irrigation in limited cases when necessary in instants where there were food remnants in the abdomen.

Our patients required significantly less parenteral analgesics that more than half of them did not ask for any pethidine injection. They had a lower visual analog pain score on postoperative days 1 and 3. This can be explained by the already existing evidence that laparoscopic correction of PPU causes less postoperative pain [11,21,26,30]. The meta-analysis published by Lau [11] reported that eight out of ten studies showed a significant reduction in dosage of analgesics required in the laparoscopic group. Also, the three studies that had included VAS pain scores showed consistently lower pain scores, as was observed in our study as well. Whether this will lead to a better quality of life for patients, especially during the first weeks after surgery still needs to be analyzed. Patients in our series who underwent laparoscopy had less postoperative pain and also a less length of hospital stay 75 ± 12.6 h. It appears that the age of PPU patients may have influenced this relatively shorter hospital stay; it was 39.5 ± 8.6 years. In most of the published series the age is increasing. This not only increases the mean hospital stay time but it may eventually represent a significant problem in the future [22,32]. One benefit of the laparoscopic procedure not often mentioned in literature pain [11] is cosmetic outcome. Nowadays patients are aware of this benefit, and sometimes this is the reason why they demand laparoscopic surgery [34].

In conclusion, the results of the current trial confirm the results of other trials that laparoscopic correction of PPU is safe, feasible for the experienced laparoscopic surgeon, and causes less postoperative pain. Operating time was less than previously reported and complications are less.

These results however, need further evaluation on bigger patients sample with more advanced age on the future studies.

## Competing interests

The authors have declared that no competing interests.
